# Spatial transcriptomics deconvolution methods generalize well to spatial chromatin accessibility data

**DOI:** 10.1093/bioinformatics/btaf268

**Published:** 2025-07-15

**Authors:** Sarah Ouologuem, Laura D Martens, Anna C Schaar, Maiia Shulman, Julien Gagneur, Fabian J Theis

**Affiliations:** School of Computation, Information and Technology, Technical University of Munich, Munich, 80333, Germany; Computational Health Center, Helmholtz Center Munich, Neuherberg, 85764, Germany; School of Computation, Information and Technology, Technical University of Munich, Munich, 80333, Germany; Computational Health Center, Helmholtz Center Munich, Neuherberg, 85764, Germany; School of Computation, Information and Technology, Technical University of Munich, Munich, 80333, Germany; Computational Health Center, Helmholtz Center Munich, Neuherberg, 85764, Germany; Computational Health Center, Helmholtz Center Munich, Neuherberg, 85764, Germany; School of Computation, Information and Technology, Technical University of Munich, Munich, 80333, Germany; Computational Health Center, Helmholtz Center Munich, Neuherberg, 85764, Germany; Institute of Human Genetics, School of Medicine and Health, Technical University of Munich, Munich, 81675, Germany; School of Computation, Information and Technology, Technical University of Munich, Munich, 80333, Germany; Computational Health Center, Helmholtz Center Munich, Neuherberg, 85764, Germany; School of Life Sciences Weihenstephan, Technical University of Munich, Munich, 80333, Germany

## Abstract

**Motivation:**

Spatially resolved chromatin accessibility profiling offers the potential to investigate gene regulatory processes within the spatial context of tissues. However, current methods typically work at spot resolution, aggregating measurements from multiple cells, thereby obscuring cell-type-specific spatial patterns of accessibility. Spot deconvolution methods have been developed and extensively benchmarked for spatial transcriptomics, yet no dedicated methods exist for spatial chromatin accessibility, and it is unclear if RNA-based approaches are applicable to that modality.

**Results:**

Here, we demonstrate that these RNA-based approaches can be applied to spot-based chromatin accessibility data by a systematic evaluation of five top-performing spatial transcriptomics deconvolution methods. To assess performance, we developed a simulation framework that generates both transcriptomic and accessibility spot data from dissociated single-cell and targeted multiomic datasets, enabling direct comparisons across both data modalities. Our results show that Cell2location and RCTD, in contrast to other methods, exhibit robust performance on spatial chromatin accessibility data, achieving accuracy comparable to RNA-based deconvolution. Generally, we observed that RNA-based deconvolution exhibited slightly better performance compared to chromatin accessibility-based deconvolution, especially for resolving rare cell types, indicating room for future development of specialized methods. In conclusion, our findings demonstrate that existing deconvolution methods can be readily applied to chromatin accessibility-based spatial data. Our work provides a simulation framework and establishes a performance baseline to guide the development and evaluation of methods optimized for spatial epigenomics.

**Availability and implementation:**

All methods, simulation frameworks, peak selection strategies, analysis notebooks and scripts are available at https://github.com/theislab/deconvATAC.

## 1 Introduction

Single-cell transcriptomics and epigenomics have significantly advanced our understanding of cellular states and their gene regulation. However, the inherent cell dissociation in these methods obscures spatial information. Spatial transcriptomics has addressed this limitation, enabling the study of cellular functions and states within their native tissue context (Marx 2021, Heumos *et al.* 2023). Further, there is an increasing interest in resolving additional cellular modalities without the need for dissecting the cellular microenvironment (Vandereyken *et al.* 2023, Klughammer *et al.* 2024, Vicari *et al.* 2024). Spatial proteomics (noa 2024) and spatial receptor sequencing (Engblom *et al.* 2023) paired with insight obtained from transcriptomics generate more complete views of local dependencies in complex tissues. Recently, the emergence of spatial chromatin accessibility technologies (Deng *et al.* 2022, Llorens-Bobadilla *et al.* 2023, Zhang *et al.* 2023, Russell *et al.* 2024, Guo *et al.* 2025) offers the potential to investigate gene regulatory processes and chromatin regulation within this spatial context.

**Figure 1.**
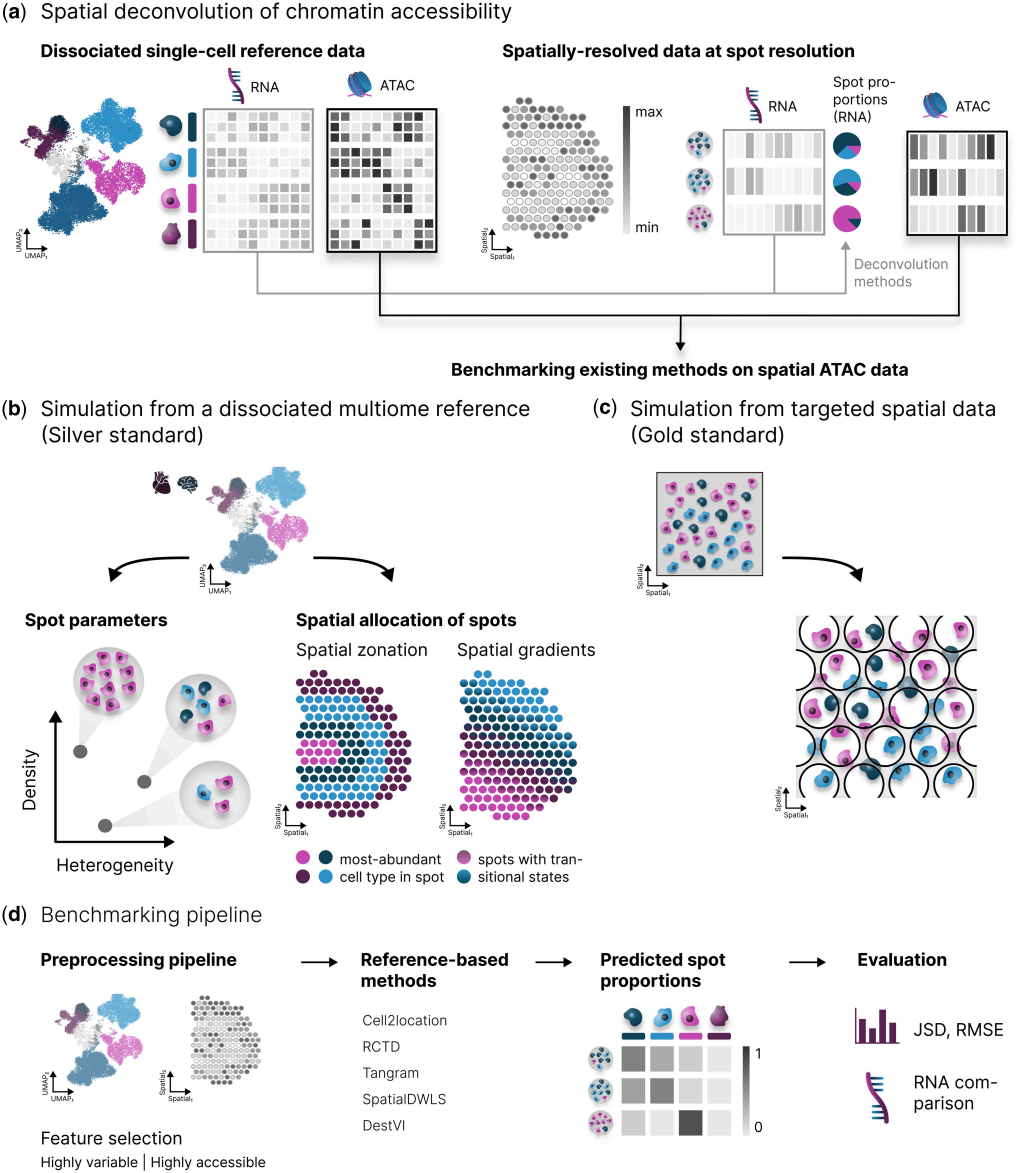
Workflow for evaluating spatial deconvolution methods developed for spatial transcriptomics data on spatial chromatin accessibility data. (a) Spatial deconvolution has been developed and successfully applied to spatial transcriptomics data at spot resolution to decompose the cell-type composition in individual spots. We evaluate deconvolution methods originally designed for transcriptomics measurements on their capabilities to deconvolve spatially-resolved chromatin accessibility data. We provide a framework to simulate spatial epigenetic data in two ways: (b) through the silver standard by leveraging dissociated references and c, through the gold standard by leveraging targeted spatial ATAC datasets. Our pipeline allows the generation of spot-based data with different cell densities, cell-type heterogeneity, and spatial distributions to accurately reflect tissue patterns observed in real datasets (b). Epigenetic information measured with targeted technologies can be aggregated to spot-based spatial data by aggregating features overlapping artificial spots (c). (d) The benchmarking pipeline incorporates different feature selection strategies and tests five deconvolution methods: Cell2location, RCTD, Tangram, SpatialDWLS, and DestVI. Performance is evaluated based on two different metrics: RMSE and JSD. Additionally, we compare the performance obtained on spatial transcriptomics measurements. Heart and brain icons were created in BioRender. Heumos, L. (2025) https://BioRender.com/d08u1k8.

Unlike spatial transcriptomics methods, spatial chromatin accessibility methods are solely sequencing-based and can be broadly categorized into two approaches. Slide-tag directly tags single-nuclei within an intact tissue using barcodes with known spatial positions, enabling subsequent single-nucleus chromatin accessibility profiling using the assay for transposase-accessible chromatin with sequencing (ATAC-seq) (Russell *et al.* 2024). However, this technology suffers from significant cell loss, hindering the detection of fine-grained spatial interactions (Russell *et al.* 2024). Alternatively, spot-based protocols (Deng *et al.* 2022, Llorens-Bobadilla *et al.* 2023, Zhang *et al.* 2023, Guo *et al.* 2025) measure gene expression or chromatin accessibility from regions containing multiple cells of potentially diverse types, resulting in composite measurements reflecting the aggregate signal. This necessitates deconvolution methods to disentangle the mixed signals and estimate cell-type proportions within each spot (Longo *et al.* 2021, Vandereyken *et al.* 2023). Several methods have been developed for spatial transcriptomics and were independently assessed in several benchmarks (Li *et al.* 2022, 2023, Yan and Sun 2023, Sang-aram *et al.* 2024). These methods typically leverage a dissociated single-cell reference to learn cell-type signatures, which are then used to deconvolve the spot-based expression vector. The methods either make no assumptions about the count distribution (Biancalani *et al.* 2021, Dong and Yuan 2021), or model the data using probabilistic models based on standard count distributions such as the negative binomial (Kleshchevnikov *et al.* 2022, Lopez *et al.* 2022) or Poisson distribution (Cable *et al.* 2022). It was shown that fragment counts in chromatin accessibility peaks exhibit similar distributions (Martens *et al.* 2024, Miao and Kim 2024), and hence, we hypothesized that deconvolution methods might be applicable to spatial epigenomic data without significant alterations of the underlying method (Fig. 1a). In this work, we benchmark five well-performing spatial transcriptomics deconvolution methods (Cell2location (Kleshchevnikov *et al.* 2022), RCTD (Cable *et al.* 2022), Tangram (Biancalani *et al.* 2021), SpatialDWLS (Dong and Yuan 2021), and DestVI (Lopez *et al.* 2022)) and assess whether they can be readily applied to emerging spatial epigenetic data. Additionally, we developed a flexible framework to simulate paired spot-based transcriptomic and accessibility data from dissociated multiome datasets (Kanemaru *et al.* 2023, Zhu *et al.* 2023) and targeted spatial multiome data (Russell *et al.* 2024) (Fig. 1b). Our simulation framework enables direct comparison of method performance across modalities. Further, we account during simulations for strong variations in tissue heterogeneity, by altering cell-type compositions, cell density and spatial zonation. Variations in these spatial structures are commonly observable in complex tissues and it has been shown that they reflect meaningful biological signals (Bhuva *et al.* 2024) and deconvolution methods should still accurately recover the cellular composition in such cases.

Spatial transcriptomics deconvolution methods are typically designed to be applied to a few thousand highly variable genes. The analysis of ATAC-seq data however commonly includes over 100,000 peaks, which raises the need for careful choice of peak selection strategies. We therefore investigate the impact of two common peak selection strategies, highly accessible or highly variable peaks.

In conclusion, this study demonstrates that certain spatial transcriptomics deconvolution methods can be successfully applied to spatial chromatin accessibility data. This work provides specific recommendations for peak selection, a robust simulation framework for generating complex spatially structured multimodal data, and establishes a performance baseline to guide the development and evaluation of methods specialized for spatial epigenomics.

## 2 Methods

### 2.1 Overview of deconvolution methods

We assessed the performance of the five deconvolution methods using SEML (Slurm Experiment Management Library) (Zügner *et al.* 2023) for experiment management. Method-specific parameter settings are detailed below.

#### 2.1.1 Cell2location

Cell2location (Kleshchevnikov *et al.* 2022) is a Bayesian probabilistic model that infers cell-type composition in spatial transcriptomics data using negative binomial regression. We used v0.1.4 and followed the guidelines from https://cell2location.readthedocs.io/en/latest/notebooks/cell2location_tutorial.html, setting detection_alpha to 20 and n_cells_per_location to 8. The means_cell_abundance_w_sf and q05_cell_abundance_w_sf posteriors were evaluated.

#### 2.1.2 DestVI

DestVI (Lopez *et al.* 2022) is a variational autoencoder-based (VAE) model employing two VAEs: a single-cell latent variable model (scLVM) and a spatial latent variable model (stLVM). The scLVM, trained on a reference single-cell RNA-seq dataset, provides a cell-type-specific latent space. The stLVM uses the trained scLVM and spatial data to predict cell-type proportions. Both models expect negative-binomially distributed expression data as input. We used scvi-tools (v1.0.3) and followed the guidelines from https://docs.scvi-tools.org/en/stable/tutorials/notebooks/spatial/DestVI_tutorial.html. We set max_epochs to 300 for scLVM and to 2000 for stLVM.

#### 2.1.3 Tangram

Tangram (Biancalani *et al.* 2021) is a deep-learning model based on non-convex optimization. The model outputs a matrix indicating the probability of each cell type in each spatial voxel. We used version v1.0.4 and followed the instructions from https://tangram-sc.readthedocs.io/en/latest/tutorial_link.html. We ran Tangram in both modes, mode=“clusters” with cluster_label=“cell_type” and mode=“cell.” In cluster mode, Tangram maps averaged cells of the same cluster instead of single cells.

#### 2.1.4 RCTD

RCTD (Cable *et al.* 2022) is a probabilistic-based method that uses maximum-likelihood estimation to predict cell-type proportions, assuming Poisson distributed counts for each spot with a log-normal prior. We used the spacexr package (v2.2.1) and followed the tutorial provided on the website: https://raw.githack.com/dmcable/spacexr/master/vignettes/visium_full_regions.html, setting doublet_mode to “full” in the run.RCTD function. We disabled feature filtering by setting gene_cutoff, fc_cutoff, gene_cutoff_reg, and fc_cutoff_reg to zero. CELL_MIN_INSTANCE and UMI_min were also set to zero.

#### 2.1.5 SpatialDWLS

SpatialDWLS (Dong and Yuan 2021) involves fitting a least squares regression model that estimates cell-type proportions by minimizing the weighted difference between the observed and predicted gene expression/accessibility values. We used the Giotto package (v4.0.4) to run SpatialDWLS. For ATAC data, we used TFIDF-normalized data and LSI for dimensionality reduction, while log-normalized data and PCA were used for RNA data. Data was clustered using the Leiden algorithm with default parameters. A signature matrix was created using makeSignMatrixDWLS with expression_values set to “normalized”, and reverse_log to TRUE for RNA and FALSE for ATAC. Deconvolution was performed with runDWLSDeconv, setting n_cell to 10.

#### 2.1.6 Baseline

As a baseline comparison, we implemented a naive deconvolution strategy that predicted only the majority cell type in each spot with a probability of 1.

### 2.2 Dataset collection and preprocessing

#### 2.2.1 Spatial chromatin accessibility datasets

**Slide-tags human melanoma:** The Russell *et al.* (2024) dataset comprises a human metastatic melanoma sample profiled using the Slide-tags method, which spatially tags and profiles single nuclei using droplet-based snATAC and snRNA-seq. The dataset includes 2529 spatially mapped nuclei from a 38.3 mm^2^ section.

#### 2.2.2 Simulation datasets

**Human heart:**  Kanemaru *et al.* (2023) measured 704,296 cells in human heart tissue samples using the 10x Genomics Multiome ATAC and Gene Expression protocol. The dataset comprises eight different anatomical regions of the human heart acquired from 25 different donors with a known history of cardiac disease or arrhythmia and an age ranging between 20 and 75 years. For our simulations of spot-based chromatin accessibility data, we used the twelve cell types originally annotated by Kanemaru *et al.* (2023). The data was obtained from the cellxgene census (Abdulla et al. 2025) and stored as a MuData object (Bredikhin *et al.* 2022, Virshup *et al.* 2023).

**Human cerebral cortex:**  Zhu *et al.* (2023) measured 45,549 nuclei in the human cortex along six developmental timepoints using the 10x Genomics Multiome ATAC and Gene Expression protocol. For our simulations of spot-based chromatin accessibility data, we used the 13 cell types originally provided by Zhu *et al.* The data was obtained from the cellxgene census (Abdulla *et al.* 2025) and stored as a MuData object (Bredikhin *et al.* 2022, Virshup *et al.* 2023).

#### 2.2.3 Peak and gene selection

For scATAC-seq data, peak selection was performed on the reference datasets by retaining either the 20,000 most accessible peaks or the 20,000 most highly variable peaks. We used ArchR’s (Granja *et al.* 2021) highly variable peak selection method which identifies highly variable peaks across clusters. In our case, the clusters were predefined by the cell-type labels. For scRNA-seq data, analyses were performed using Scanpy (Wolf *et al.* 2018, Virshup *et al.* 2023) (v1.9.5). Raw gene expression counts were normalized using Scanpy’s normalize_total function, followed by log(expression + 1) transformation. The 4000 most highly variable genes were then selected using the “seurat” flavor. The same selected feature space was then used for the spatial datasets.

### 2.3 Simulation of spatial datasets

For systematic evaluations and comparisons of the described methods, we generated two different types of benchmarking datasets. We refer to them as gold standard and silver standard. Silver standard benchmarking datasets were simulated from the heart (Kanemaru *et al.* 2023) and brain (Zhu *et al.* 2023) single-cell multiome datasets using a simulation framework that is conceptually inspired by RCTD (Cable *et al.* 2022) and Stereoscope (Andersson *et al.* 2020), while incorporating some key modifications. Similar to these methods, our framework generates spot-based data by randomly sampling a specified number of cells from the dissociated single-cell reference and aggregates their expression and accessibility profiles. Departing from these methods, we modeled both tissue heterogeneity (number of cell types per spot) and spot cell density using a Poisson-Gamma distribution, which has been shown to provide a more realistic representation of cell-type compositions than uniform sampling (Liu *et al.* 2023). Cell types were sampled with equal probability, and the overdispersion parameter of the Poisson-Gamma distribution was fixed at 20 while the mean was varied to control the number of cells and cell types in each simulated spot. Furthermore, we incorporated spatially varying cell-type compositions, which we refer to as spatial zonation (Fig. 1b), a feature absent in previous benchmarks, simulating both stripes and circles of differing compositions. The gold standard simulated benchmarking dataset was generated from the Russell *et al.* (2024) targeted spatial multiome dataset by aggregating gene expression and fragment counts from cells to larger spot-like areas, referred to as pseudo spots (Fig. 1c). Simulation parameters are summarized in Table 1. By employing these diverse simulation strategies, we argue that no spatial deconvolution method has an unfair advantage or benefits from the underlying distribution assumptions.

**Table 1.** Parameters used for generating simulated datasets.

Dataset	Reference	Number of spots	Zonation type	Number of regions	Mean number of cell types per spot	Mean number of cells per spot
Heart/Brain 1	Heart/Brain multiome	1000	Uniform	1	3	5
Heart/Brain 2	Heart/Brain multiome	1000	Uniform	1	10	15
Heart/Brain 3	Heart/Brain multiome	1000	Zonated	4	10, 5, 10, 5	15, 10, 15, 5
Heart/Brain 4	Heart/Brain multiome	1000	Zonated	4	3, 5, 3, 5	15, 10, 15, 5
Russell	Slide-tags	360	Artificial spots	N/A	N/A	N/A

Note: This table summarizes the key parameters used to generate the simulated benchmarking datasets from dissociated (Heart/Brain) and targeted spatial data (Russell).

Further, we assessed Tangram’s stability in cell mode by simulating reference datasets of varying sizes using the dissociated human heart tissue dataset due to observed performance differences for varying reference dataset sizes. The smallest reference dataset contained only the cells that were used to generate spots in a corresponding spatial simulation. This smallest reference dataset was then incrementally expanded by adding cells, without replacement, sampled from the full human heart reference dataset. The size of the expanded reference was systematically increased by factors of 2, 4, 8, and 16 times the size of the smallest reference.

### 2.4 Evaluation metrics

Deconvolution performance was evaluated by comparing predicted cell-type proportions to the true cell-type proportions using two primary metrics: root mean squared error (RMSE) and Jensen–Shannon divergence (JSD). RMSE quantifies the absolute difference between predicted and true proportions, while JSD measures the similarity between the distributions of predicted and true cell-type proportions. We also assessed the agreement between predicted and true majority cell-type assignments using the Normalized Mutual Information (NMI), a measure of similarity between two data clusterings. Furthermore, we assessed the ability of the methods to recover rare cell types using the F1 score, which computes the harmonic mean of precision and recall for the detection of those cell types.

## 3 Results

### 3.1 Evaluation pipeline

In order to efficiently benchmark different methods and assess the impact of individual parameter choices, we designed an end-to-end benchmarking pipeline. Our pipeline compares five deconvolution methods on a set of two differently designed simulated datasets and reports a set of pre-selected metrics (Fig. 1d). Specifically, we simulate spatial spots based on dissociated multiome datasets by incorporating realistic tissue heterogeneity and spatial variability (Fig. 1b), and additionally, by simulating a spatial dataset derived from a targeted spatial multiome experiment (Fig. 1c). Spot-based spatial transcriptomics data typically measures up to 30,000 genes, but commonly only a few thousand highly variable genes are then used during spatial deconvolution. In contrast, ATAC-seq data measures over 100,000 peaks and feature selection can either be performed based on highly variable peaks or highly accessible peaks. For the methods to scale, we therefore investigated the impact of feature selection on the performance of deconvolution methods for spatial chromatin accessibility data. We compared two common peak selection strategies: selecting highly accessible peaks, which represent common and robust signals, and selecting highly variable peaks, which should be informative for distinguishing cell types (Fig. 1d). Accuracy was evaluated using RMSE and JSD between predicted and true cell-type proportions (Fig. 1d). The multiome data enabled direct comparison of RNA and ATAC deconvolution performance.

### 3.2 Performance evaluation of deconvolution methods

**Figure 2.**
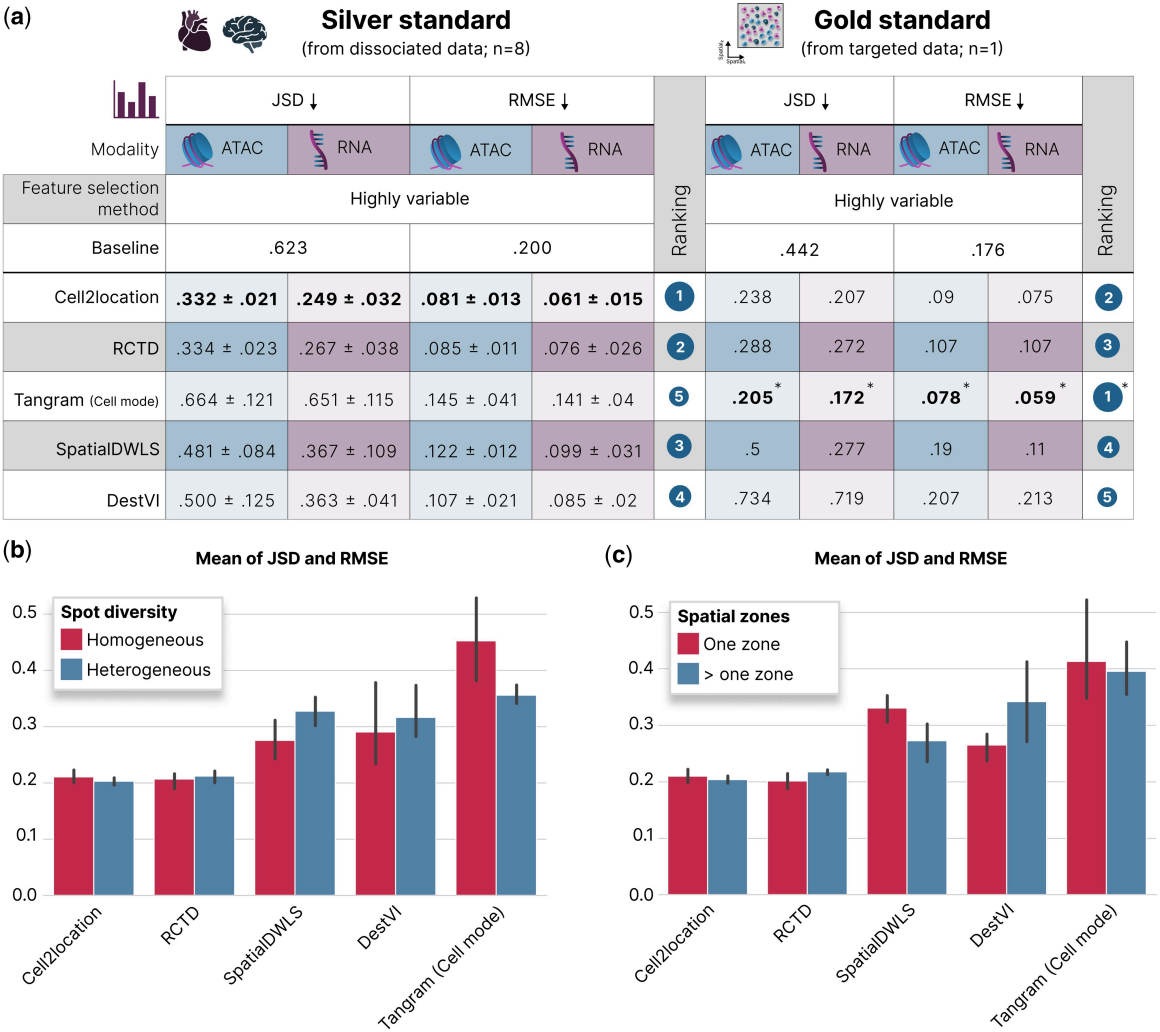
Performance evaluation of spatial deconvolution methods on simulated data. (a) Performance of the deconvolution methods on simulated datasets derived from the silver standard simulations and the gold standard simulations. JSD and RMSE are reported for both ATAC-seq and matched RNA-seq data on highly variable features, which consistently showed higher performance compared to highly accessible features (Supplementary Table S1). Lower JSD and RMSE values indicate better performance. For silver standard evaluations, the table reports the mean and standard deviation across all simulations (heart and brain, *n* = 8). For gold standard evaluations, the table reports the respective metric (*n* = 1). Method rankings are based on the mean JSD and RMSE of the ATAC deconvolution across both feature selection methods only. Tangram Cell Mode results for targeted data have inflated scores (see main text) as indicated by the asterisk (*). Tangram Cluster mode consistently showed lower performance and is omitted here (Supplementary Table S1). (b) Mean of JSD and RMSE for each method on the ATAC modality and using highly variable features on homogeneous (few different cell types per spot, *n* = 4) and heterogeneous (many different cell types per spot, *n* = 4) simulated datasets. (c) Mean of JSD and RMSE for each method on the ATAC modality and using highly variable features on simulated datasets with a single (*n* = 4) and multiple zones (*n* = 4), each zone having distinct cell-type compositions. Error bars indicate 95% confidence intervals using bootstrapping. Heart and brain icons were created in BioRender. Heumos, L. (2025) https://BioRender.com/d08u1k8.

As the methods presented in this work are primarily designed for spatial transcriptomics data, we compared whether there is a significant drop in deconvolution performance when instead leveraging ATAC-based spatial data (Fig. 2a and Supplementary Table S1). For Tangram we evaluated both model-inherent modes, the cell and cluster mode, which differ in the way they assign cell types to spots (Methods; see Supplementary Table S1 for cluster mode results). Despite the inherent sparsity of ATAC-seq data, Cell2location and RCTD demonstrated robust performance on the chromatin accessibility modality. They ranked highest according to the mean JSD and RMSE across both simulation strategies (Fig. 2a and Supplementary Table S1). This is in concordance with previous benchmarks where these methods ranked top on spatial transcriptomics data (Li *et al.* 2022, Yan and Sun 2023, Sang-aram *et al.* 2024). However, while overall results were comparable, RCTD’s and Cell2locations’ performance on ATAC was still slightly lower than the RNA-based deconvolution, suggesting that there is potential for further refinement of existing or development of new methods that are specifically optimized for ATAC-based spatial data. SpatialDWLS and DestVI performed worse on the data simulated from dissociated data, scoring only slightly better than the baseline for the ATAC modality. The performance of DestVI was not consistent across all datasets, showing even further reduced accuracy on targeted-data simulations which was also noted in previous studies (Sang-aram *et al.* 2024). For Tangram we observed a variable deconvolution performance. On simulations obtained with dissociated data, both Tangram’s cell and cluster modes generally performed no better than the baseline, except for marginal improvement using the cluster mode with RNA-seq data (Supplementary Table S1). In contrast, on simulations obtained with targeted spatial data, the cell mode achieved unexpectedly high performance, while the cluster mode remained poor. We hypothesized that this disparity might be due to Tangram’s cell-to-spot mapping strategy. For reference datasets as constrained in composition as for the targeted simulation (2529 cells that are also part of the spatial slide), we expect the direct cell to spot mapping to be easier. As we observed this phenomena only for Tangram and not for any other spatial deconvolution method, we simulated different reference compositions using the heart tissue dataset (Kanemaru *et al.* 2023) and assessed the performance of Tangram to validate our hypothesis (Methods). We observed that Tangram’s performance was optimal when the reference dataset composition and size matched with the size of the spatial dataset and deteriorated when more cells were present in the reference dataset (Supplementary Fig. S1), suggesting that Tangram’s performance is sensitive to the reference.

Across all methods, we observed a significant difference in performance based on the feature selection strategy used. Specifically, the selection of highly variable peak selection consistently outperformed highly accessible peak selection (*P* = 0.007, two-sided Wilcoxon test, *n* = 9; Supplementary Table S1), highlighting the importance of appropriate feature selection.

We then assessed the influence of simulation parameters, namely cell-type heterogeneity and spatial zonation (Fig. 2b and c). Cell2location and Tangram increased in performance with higher cell-type heterogeneity, whereas the other methods decreased in performance (Fig. 2b). While Cell2location and RCTD were relatively robust to cell-type heterogeneity, SpatialDWLS and DestVI showed greater performance degradation on heterogeneous dataset. Tangram also showed a substantial performance drop, though in the opposite direction from SpatialDWLS and DestVI, showing better performance on heterogeneous datasets. This observed effect on the Tangram method was confirmed to not be an artifact of the full reference being used for deconvolution, by showing the same behaviour when a reduced reference was used. Analyzing performance across different zonation patterns (Fig. 2c) showed that Cell2location outperformed RCTD in scenarios with spatially varying cell-type compositions, achieving lower mean RMSE and JSD (0.2037 vs. 0.2174). SpatialDWLS and DestVI exhibited greater sensitivity to spatial zonation compared to Cell2location and RCTD, with SpatialDWLS performing better on datasets with multiple zones and DestVI demonstrating higher accuracy on datasets with fewer zones. Overall, Cell2location and RCTD showed consistently robust performance in our simulations, while Tangram’s accuracy was sensitive to the reference dataset.

### 3.3 Deconvolution of a human metastatic melanoma dataset

**Figure 3.**
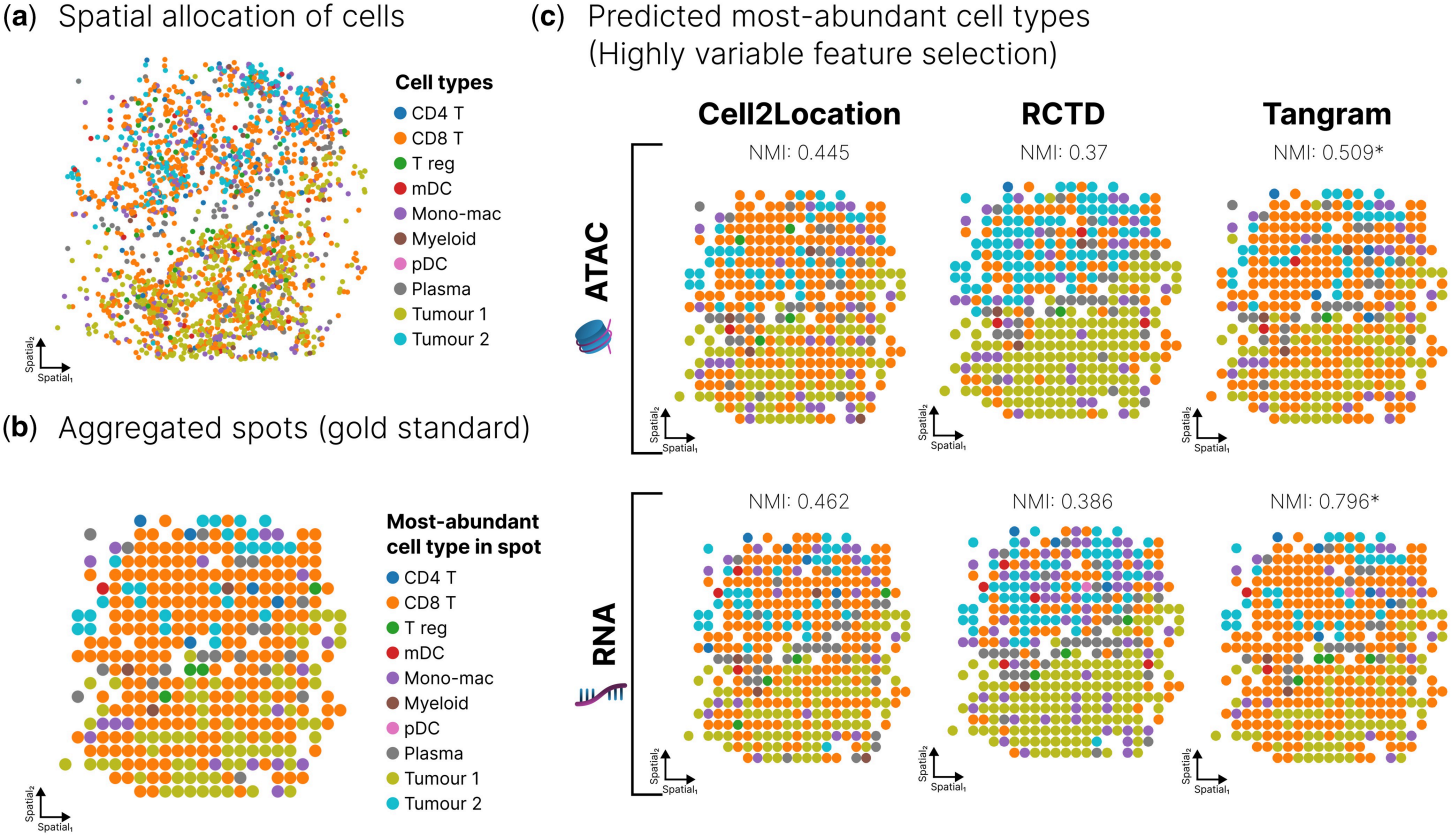
Spatial deconvolution using both RNA-based and ATAC-based deconvolution on a spatial human metastatic melanoma multiome dataset. (a) Spatial allocation of cells in the original Russell *et al.* dataset, colored by cell type. (b) Spatial plot of simulated spots generated by aggregating cells in spatial regions and colored by the dominant cell type. (c) Predicted dominant cell type per spot using Cell2location, RCTD and Tangram on both ATAC-seq and RNA-seq data with highly variable feature selection. T CD4: CD4-positive T cell; T CD8: CD8-positive T cell; T reg: regulatory T cell; mDC: myeloid dendritic cells; mono-mac: monocyte-derived macrophages; pDC: plasmacytoid dendritic cell.

We showcase the deconvolution results of the three best performing methods, Tangram, Cell2location and RCTD, on the Russell *et al.* dataset (Russell *et al.* 2024), a spatially resolved human metastatic melanoma sample profiled with both RNA-seq and ATAC-seq. This dataset exhibits two spatially distinct tumor subpopulations (denoted as tumor 1 and tumor 2) with extensive immune cell infiltration (Fig. 3a). We generated synthetic spots by aggregating expression and accessibility profiles of cells within defined spatial regions (Fig. 3b). Figure 3c compares the predicted dominant cell type for each spot using Tangram, Cell2location and RCTD across data modalities. ATAC-based deconvolution showed similar, but slightly lower performance in spatial localization of major cell types compared to RNA-based deconvolution (NMI: 0.445 vs. 0.462 for Cell2location). This performance reduction is partly explained by RNA-based deconvolution’s greater ability to resolve rare cell types. For example, we observed that RNA-based deconvolution achieved higher F1 scores than ATAC-based deconvolution for CD4+ T cells (0.6 vs. 0.2), regulatory T cells (0.4 vs. 0.25), and myeloid dendritic cells (0.2 vs. 0.0). Cell2location outperformed RCTD in this heterogeneous tissue by accurately predicting lower proportions of tumor cells relative to CD8+ T cells, also shown by a higher NMI score. These results, combined with our benchmarking analysis (Fig. 2), suggest that while RCTD and Cell2location exhibit comparable performance on many simulated datasets, Cell2location demonstrates superior performance in complex tissues with substantial variations in cell-type composition. Finally, as discussed earlier, Tangram demonstrated strong performance on this specific dataset. However, its performance, particularly given the very small reference dataset, should be interpreted cautiously.

## 4 Discussion

This study evaluated the applicability of five established spatial deconvolution methods (Biancalani *et al.* 2021, Dong and Yuan 2021, Cable *et al.* 2022, Kleshchevnikov *et al.* 2022, Lopez *et al.* 2022), originally developed for spatial transcriptomics, to the deconvolution of emerging spatial chromatin accessibility data. To this end, we developed a flexible simulation framework that generates both transcriptomic and chromatin accessibility data from dissociated single-cell multiome datasets and targeted spatial multiome datasets to directly compare the performance across modalities. This framework additionally allowed us to assess the impact of various factors, including cell density, cell-type heterogeneity, tissue zonation, and peak selection strategies, on the performance of deconvolution methods applied to spatial chromatin accessibility data. Our evaluation showed that Cell2location and RCTD can effectively deconvolve spatial chromatin accessibility data, exhibiting the highest performance among the tested deconvolution methods. This observation also aligns with prior benchmarks on spatial transcriptomics data (Li *et al.* 2022, Yan and Sun 2023, Sang-aram *et al.* 2024), highlighting the general robustness of these methods and the transferability of their model assumptions to chromatin accessibility data. Notably, the two methods were also robust to variations in cell density, cell-type heterogeneity, and tissue zonation, with Cell2location performing slightly better in heterogeneous settings. SpatialDWLS and DestVI exhibited performance close to a simple baseline for ATAC-seq data, suggesting a limited applicability of these methods to this new data modality. Tangram showed variable performance on both modalities that was especially impacted by the composition of the reference dataset. Specifically, when Tangram was applied to spot-based data using a reduced reference, it performed well. However, its performance deteriorated with larger and more complex reference datasets. We therefore recommend carefully considering the reference dataset size when using Tangram for deconvolution of spatial chromatin accessibility data. We generally found that selecting highly variable features improved performance across all methods compared to selecting the most accessible peaks, indicating the importance of appropriate feature selection for chromatin accessibility data. Although Cell2location and RCTD achieved performance levels on ATAC-based deconvolution that were comparable to those observed for RNA-based deconvolution, the latter still worked better, especially at resolving rare cell types. This performance discrepancy likely stems from two primary constraints: the inherent sparsity of ATAC-seq data itself, a factor we suspect mirrors the established sensitivity of spatial RNA-seq deconvolution to sparsity (Li *et al.* 2022) and the fact that current methods are designed for RNA-seq-based deconvolution. For instance, Cell2location and RCTD model overdispersed counts, while chromatin accessibility peak counts are often accurately modeled using a Poisson distribution (Martens *et al.* 2024). One evident limitation of our pipeline is the limited availability of gold standard datasets measuring chromatin accessibility and transcriptomics in space at single-cell resolution. Hence, our work is currently only considering one gold standard simulation dataset. However, future targeted spatially resolved chromatin accessibility datasets can easily be added to our pipeline. Despite these limitations, our results demonstrate that direct application of existing spatial transcriptomics deconvolution methods to spatial chromatin accessibility data is possible. This study additionally provides a simulation framework to simulate realistic data and establishes a performance baseline for spatial epigenomic deconvolution, against which future methods can be compared.

## Supplementary Material

btaf268_Supplementary_Data

## Data Availability

The human heart and cerebral cortex data was obtained from the cellxgene census at https://cellxgene.cziscience.com/collections. The Slide-tags dataset was downloaded from the Broad Institute Single Cell Portal under the accession number SCP2176.
