# Supplementary material for: Spatial transcriptomics deconvolution methods generalize well to spatial chromatin accessibility data
Source: Bioinformatics. 2025 Jul 15;41(Suppl 1):i314–22. doi: 10.1093/bioinformatics/btaf268 (PMC12261446; doi:10.1093/bioinformatics/btaf268)
Supplement: btaf268_Supplementary_Data [file btaf268_supplementary_data.zip › btaf268_Supplementary_Data/Theis.191.supplementary.fig.1.pdf]

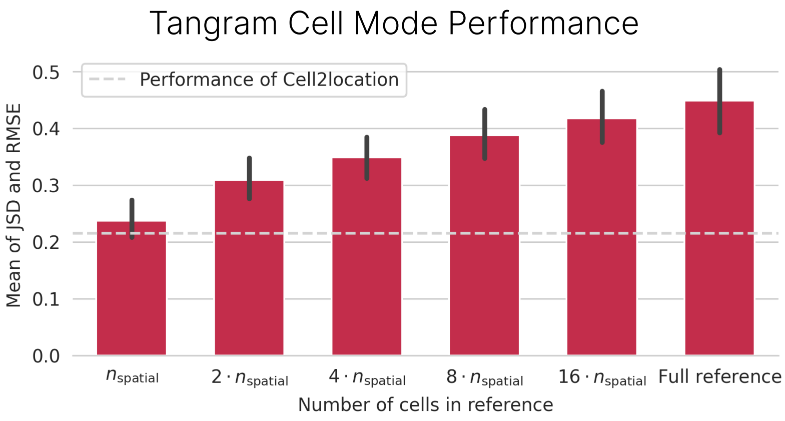

Supplementary Figure 1: Tangram cell mode performance across varying reference dataset compositions. The mean JSD and RMSE are shown for Tangram's cell mode as the reference dataset size increases relative to the size of the corresponding simulated spatial dataset ($n_{spatial}$). The "Full reference" represents the entire human heart reference dataset. Error bars indicate 95% confidence intervals using bootstrapping (n=4). The dashed line indicates the performance of the best performing method (Cell2location). The variable sized reference datasets were simulated by incrementally expanding the smallest reference set, which contained only the cells used to create the corresponding spatial dataset, by adding cells from the full dataset in multipliers of 2, 4, 8 and 16.
